# Ti^3+^ in corundum traces crystal growth in a highly reduced magma

**DOI:** 10.1038/s41598-020-79739-4

**Published:** 2021-01-28

**Authors:** Beñat Oliveira, William L. Griffin, Sarah E. M. Gain, Martin Saunders, Jeremy Shaw, Vered Toledo, Juan Carlos Afonso, Suzanne Y. O’Reilly

**Affiliations:** 1 ARC Centre of Excellence for Core to Crust Fluid Systems (CCFS) and GEMOC, Earth and Environmental Sciences, Macquarie University, Sydney, NSW 2109 Australia; 2 Centre for Microscopy, Characterisation and Analysis, The University of Western Australia, Crawley, WA 6009 Australia; 3 Shefa Gems (A.T.M.) Ltd., 4210602 Netanya, Israel

**Keywords:** Solid Earth sciences, Geochemistry, Mineralogy, Petrology, Volcanology

## Abstract

Aggregates of corundum crystals with skeletal to hopper morphology occur in pyroclastic rocks erupted from Cretaceous basaltic volcanoes on Mt Carmel, N. Israel. The rapid growth of the crystals trapped volumes of the parental Al_2_O_3_-supersaturated melt; phenocrysts of tistarite (Ti_2_O_3_) in the trapped melts indicate crystallization at oxygen fugacities 6–7 log units below the Iron-Wüstite buffer (*f*O_2_ = ΔIW − 6 to − 7), induced by fluxes of mantle-derived CH_4_-H_2_ fluids. Cathodoluminescence images reveal growth zoning within the individual crystals of the aggregates, related to the substitution of Ti^3+^ in the corundum structure. Ti contents are < 0.3 wt% initially, then increase first linearly, then exponentially, toward adjacent melt pockets to reach values > 2 wt%. Numerical modelling indicates that the first skeletal crystals grew in an open system, from a moving magma. The subsequent linear increase in Ti reflects growth in a partially closed system, with decreasing porosity; the exponential increase in Ti close to melt pockets reflects closed-system growth, leading to dramatic increases in incompatible-element concentrations in the residual melts. We suggest that the corundum aggregates grew in melt/fluid conduits; diffusion modelling implies timescales of days to years before crystallization was terminated by explosive eruption. These processes probably operate in explosive volcanic systems in several tectonic settings.

## Introduction

The oxygen fugacity (*f*O_2_) of Earth’s upper mantle can be estimated from the mineral assemblages of mantle-derived xenoliths and of magmas derived by partial melting of the mantle^1,2^. These estimates range from more oxidising than the quartz-fayalite-magnetite (QFM) buffer to near the iron-wüstite (IW) buffer. Recent advances using the Fe^3+^/Fe^2+^ ratios of mantle-derived garnets are adding finer detail, showing that the estimated *f*O_2_ of the subcontinental lithospheric mantle (SCLM) broadly decreases with depth relative to QFM, but rarely drops below the IW buffer^3,4^. Several authors^5,6^ have argued that the deeper levels of the mantle may be metal-saturated, and thus are buffered at or near IW. Under such conditions, any C–O–H fluid present will be dominated by CH_4_ + H_2_, and this appears to be confirmed by the discovery of inclusions of metallic phases + CH_4_ + H_2_ in diamonds derived from the deep upper mantle^7^.

However, several lines of evidence indicate that volumes with much lower *f*O_2_ exist locally within the mantle. One is the common occurrence of moissanite (SiC) in kimberlites^8,9^, which implies *f*O_2_ 6–8 orders of magnitude below the IW buffer^10^. SiC and other phases indicative of very low *f*O_2_ (nitrides, silicides, carbides) have been reported from peridotites and chromitites in ophiolites from Tibet, Myanmar, and the Polar Urals^11–13^. Some have argued that the SiC in kimberlites and ultramafic rocks formed by carboreduction at high temperature (*T*) near the surface^14^ or by low-pressure (*P*), low-*T* reduction during serpentinization^15^. However, the occurrence of moissanite as inclusions in diamond^16^, and the common association of SiC with Fe-Ti silicides^17^ indicates that SiC can crystallize at high temperatures in the deep lithospheric mantle.

The occurrences in kimberlites and ophiolites are known almost entirely from mineral separates, providing little context for the phases of interest^18^. This leaves the problems of how to produce such reducing conditions, the time scales on which they develop and survive and how to preserve the reduced assemblages from oxidation by the surrounding mantle^10,15^. More insights into this problem are now coming from a suite of super-reduced mineral assemblages, crystallized from high-*T* melts trapped in corundum aggregates (microxenoliths) ejected from Cretaceous volcanoes in northern Israel^17,18^. These in situ assemblages illustrate the processes that can produce localized super-reducing conditions in the uppermost mantle.

In this report we show that high contents of Ti in corundum from this area reflect the substitution of Ti^3+^ for Al^3+^ under highly reducing conditions, and use the zoning patterns of Ti^3+^ and other minor elements in corundum to constrain the crystallization processes and the nature of the environment in which these aggregates grew.

## Background: geological setting and sampling

The material described here comes from Late Cretaceous pyroclastic basaltic volcanoes on Mount Carmel in Israel, and from related alluvial deposits, mainly in the Pliocene–Quaternary terrace gravels of the Kishon River (southern Galilee; Fig. SD-1). The volcanism was mainly explosive and has been ascribed to “hot-spot” or “plume-related” magmatism in an intraplate setting^19^. The eruptions continued over a period of *ca* 13 m.y. (98–85 Ma^20,21^), and over an area of at least 150 km^2^, and the samples described here are derived from the tuffs of several eruptive centers, and associated alluvial deposits. Detailed descriptions of the geological setting have been provided by^21–23^.

Heavy-mineral concentrates from the Cretaceous tuffs have yielded Cr-diopside, olivine, orthopyroxene, garnet and chromite derived from garnet-spinel peridotites and pyroxenites, as well as megacrysts of Mg-ilmenite, amphibole, zircon and diopside and less-common minerals such as moissanite (crystals to > 4 mm), rutile, hibonite and corundum^21,23,24^. In addition to gem-quality sapphire and ruby, the erupted material contains abundant xenocrysts of “Carmel Sapphire” up to 2.5 cm in diameter (> 30 carats).

## Background: the Mt Carmel super-reduced assemblages

**Figure 1:**
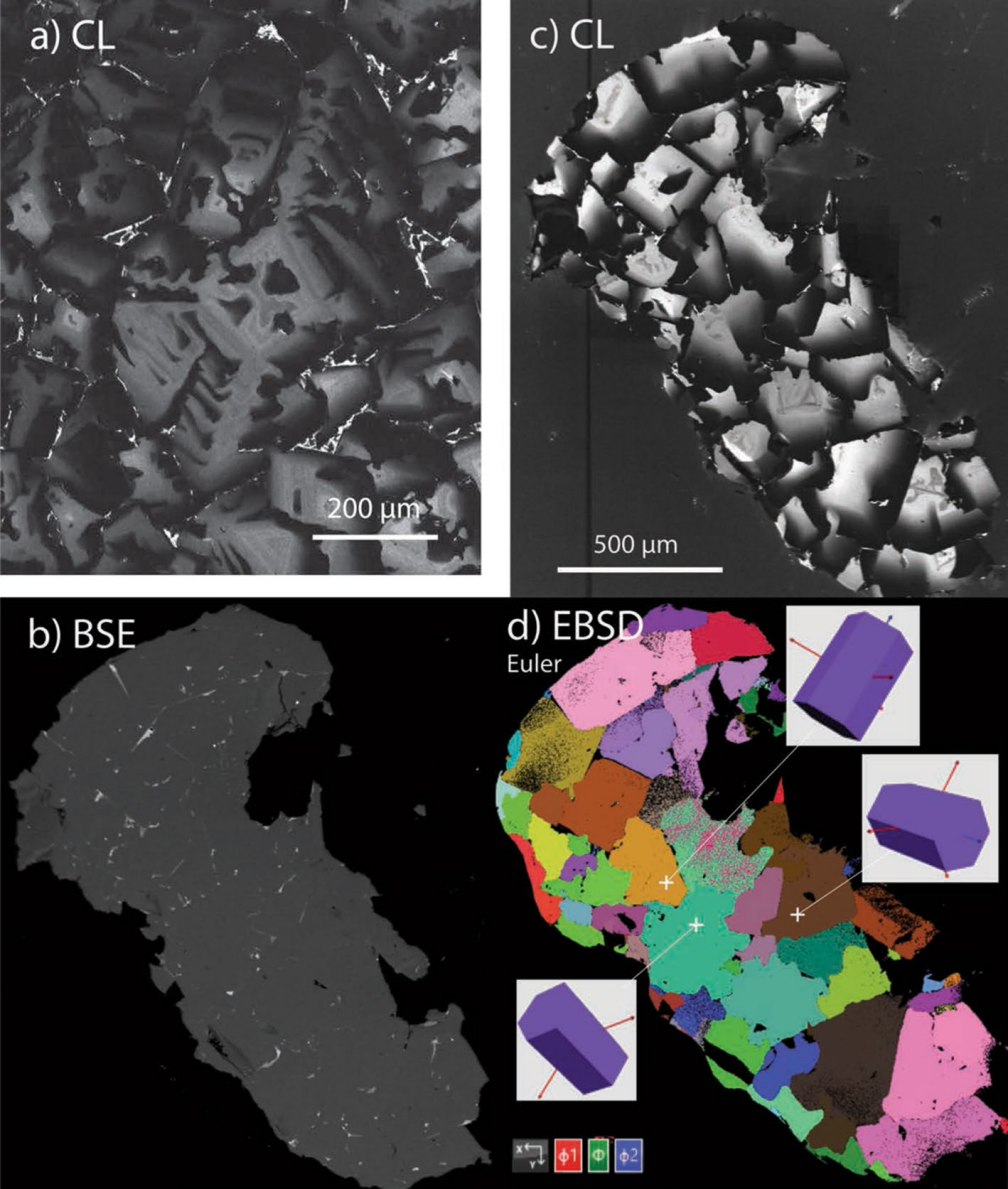
(**a**) CL image of skeletal corundum in the core of a Carmel Sapphire crystal; (**b**) BSE, (**c**) CL and (**d**) EBSD images showing growth of Mt Carmel “non-gem” corundum (Carmel Sapphire). Note skeletal structures in the bright cores of most crystals, surrounded by smoother zoning. Prisms in (**c**, **d**) show the orientation of single crystals or of most crystals in small aggregates that make up the larger aggregate.

Back-scattered electron and cathodoluminescence (CL) images of Carmel Sapphires (Fig. 1) show that most samples are aggregates of crystals with skeletal to hopper forms, which have trapped pockets of their parental melts in a complex 3-dimensional network [^17^; Fig. SD2]. The melts are now preserved as Ca-Al-Si-O glasses (confirmed as amorphous by TEM analysis and Raman spectroscopy). Skeletal/hopper crystallization is typical of rapid growth from supersaturated melts. The mineral parageneses in the melt pockets allow a detailed reconstruction of the conditions under which the corundum crystallized. The appearance of dmisteinbergite (a polymorph of anorthite, identified by Raman spectroscopy) as a quench phase in the glasses defines the peritectic reaction corundum + melt → anorthite, requiring pressures > 0.9 GPa and T > 1400 °C^25^. High levels of Al-Ti substitution for Mg in spinels are consistent with temperatures ranging from ca 1500 °C to 1200 °C, as are the compositions of immiscible Fe-Ti-Si melts found in many melt pockets^17,18^. These conditions imply crystallization in the uppermost mantle, near the crust-mantle boundary beneath the area. The presence of glass suggests that crystallization was interrupted by entrainment in the rapidly ascending basaltic magmas; this was accompanied by extensive brecciation, the veins filled with a mixture of crystal fragments and amorphous carbon.

**Figure 2:**
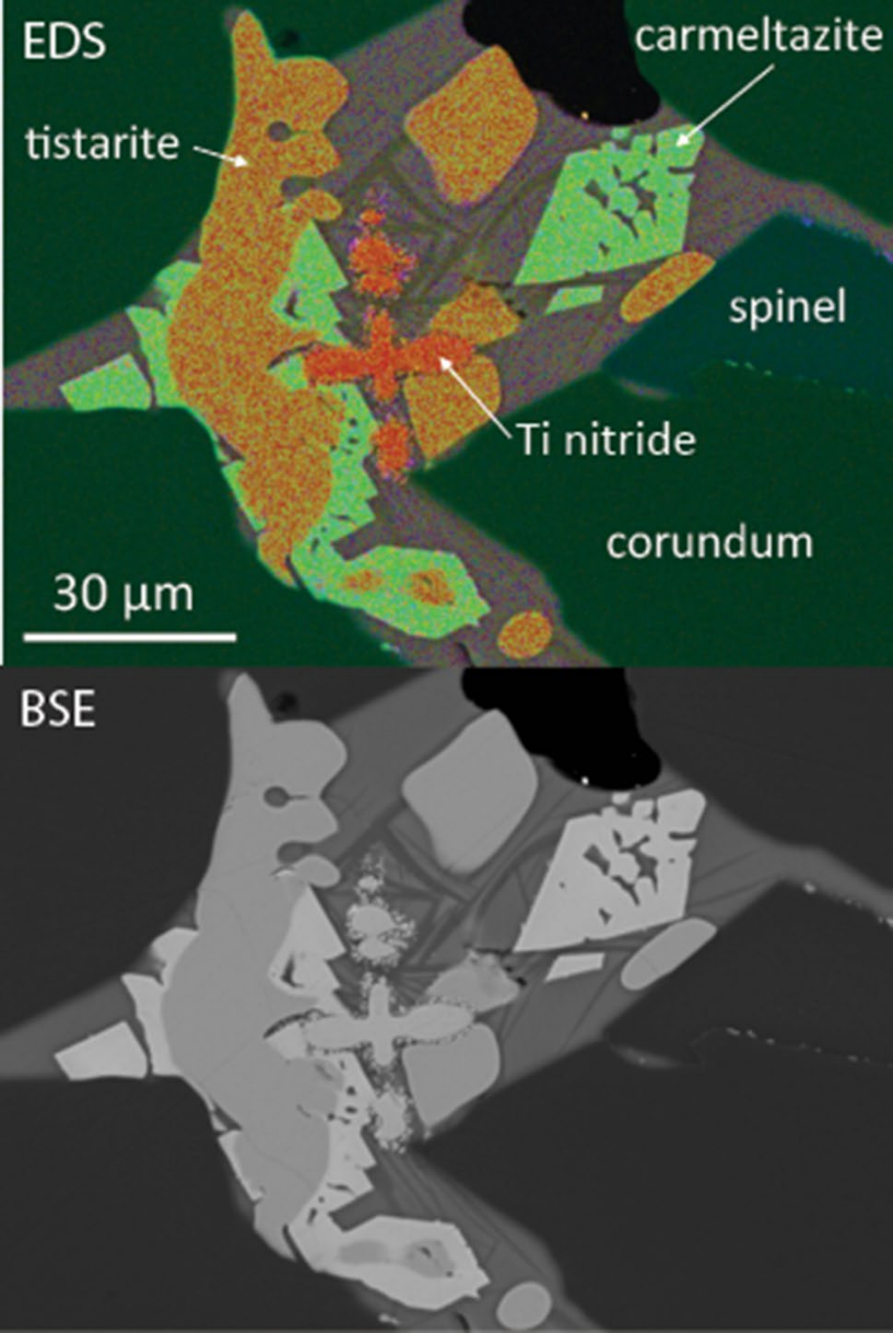
Melt pocket in corundum, showing the typical paragenesis of tistarite (Ti_2_O_3_) and carmeltazite (ZrAl_2_Ti_4_O_11_) in glass with quench crystals of dmisteinbergite (CaAl_2_Si_2_O_8_).

The first phase to crystallise from the trapped silicate melts typically is tistarite (Ti_2_O_3_), which requires *f*O_2_ at least 7 log units below the IW buffer (ΔIW − 7^17^). This *f*O_2_ also is close to the stability limit of the assemblage SiO_2_ + C, which is consistent with the appearance of moissanite (SiC) as inclusions in the corundum. The tistarite is typically resorbed and overgrown by carmeltazite (ZrAl_2_Ti_4_O_11_^26^; Fig. 2). Further reduction is implied by the presence of Ti^2+^-bearing phases such as TiB_2_^38^.

The melt pockets also contain a range of Fe-Ti-Si (P, Zr) alloys with globular forms indicating that they represent immiscible melts, which also crystallized abundant khamrabaevite (TiC) and lesser moissanite^17^.

We have previously suggested^17,18^ that the Al_2_O_3_-supersaturation required to produce the rapid crystallization of corundum was induced by progressive reaction between differentiated mafic melts and fluids dominated by CH_4_ + H_2_, derived from a metal-saturated mantle. This argument is supported by the abundance of carbon in the system, and the requirement for the presence of H_2_ to reach the lowest recorded values of *f*O_2_ (ΔIW − 10, equivalent to the early solar nebula) in coarse-grained hibonite-grossite-vanadium xenoliths found in the same tuffs^27,28^.

## Results

### Compositional variation in corundum

A striking feature of the Carmel Sapphire is its intense cathdoluminescence (CL), which typically is a bright pink. The growth histories of the corundum crystals are recorded by striking variations in their CL patterns (Fig. 1). Comparison of CL images with electron back-scattered diffraction (EBSD) imaging shows that groups of two or more skeletal/hopper crystals can show subparallel axes, while individual multi-crystal domains can have a range of orientations (Fig. 1). The CL is progressively extinguished toward interstitial melt pockets or toward volumes of melt trapped within the cavities of skeletal crystals. Electron microprobe (EMP) analyses (Table SD1) show that the CL intensity is inversely correlated with the concentration of Ti, which ranges up to 2.5 wt%, or 0.56 at.%. Such high concentrations are unusual; most known corundum, including intensely coloured varieties, contains < 0.5 wt% Ti^29^.

**Figure 3:**
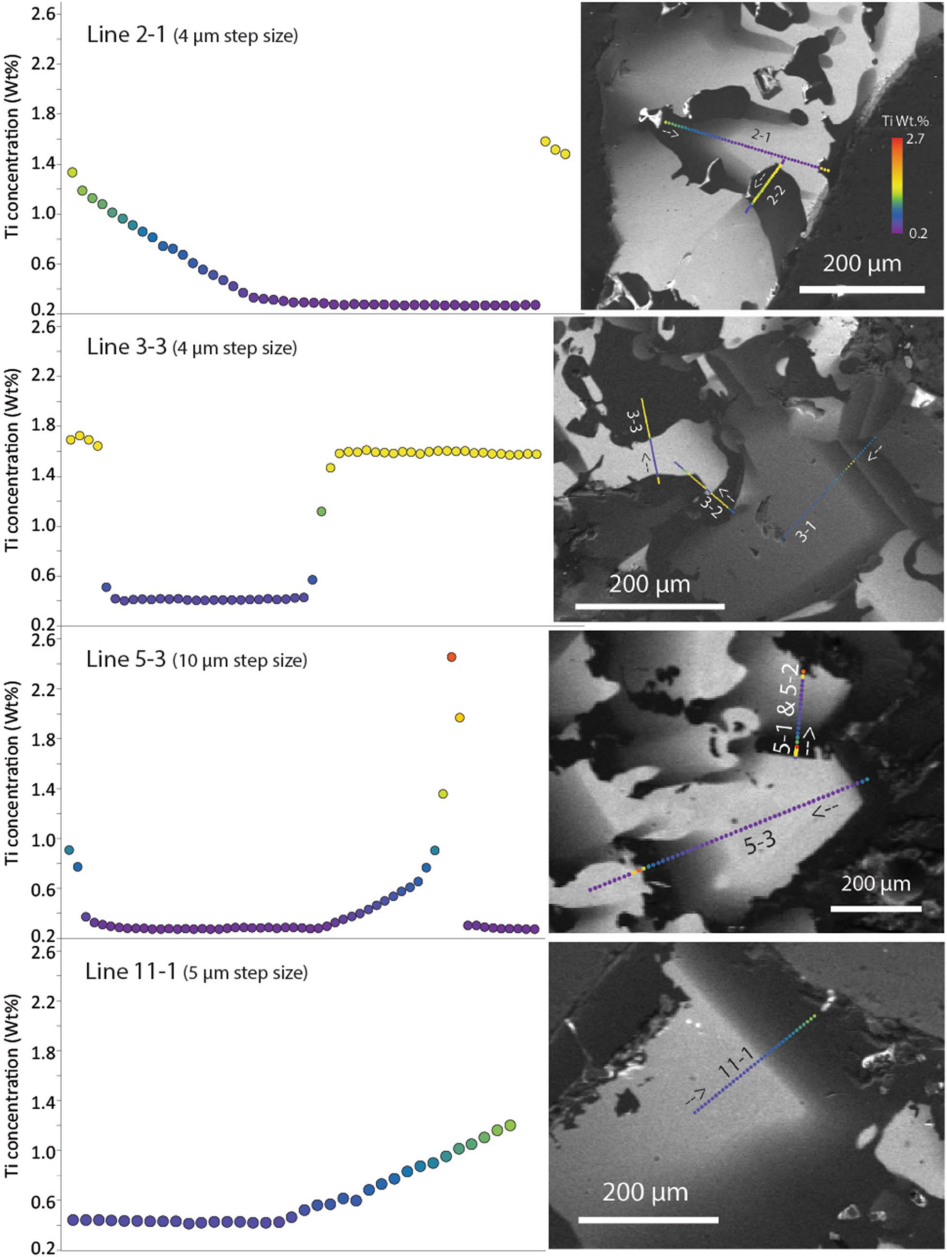
Montage of EMP traverses discussed in text, with CL images showing locations.

EMP traverses (Fig. 3) that map Ti content across crystals, toward visible melt pockets, typically show patterns like grains 2-1 and 11-1, in which Ti contents start at low levels (0.2–0.4 wt% Ti), increase only weakly as the crystal grows, and subsequently start rising linearly toward the melt pocket. Others, like 5-3, show a similar pattern until very close to the melt pocket, then increase more rapidly. Dark-CL zones also may abut light-CL zones sharply, with no visible gradient. Such contacts tend to occur along the sides of corundum prisms, or where zones of dark CL appear to intrude or replace the lighter-CL corundum. An example is traverse 3-3, which shows plateaus at 0.4 wt% and 1.6 wt% Ti, separated by short segments with very steep diffusional profiles. These sharp contacts may reflect loci where melts have moved along external or internal boundaries but are no longer present, having been displaced by the continued growth of the corundum.

**Figure 4:**
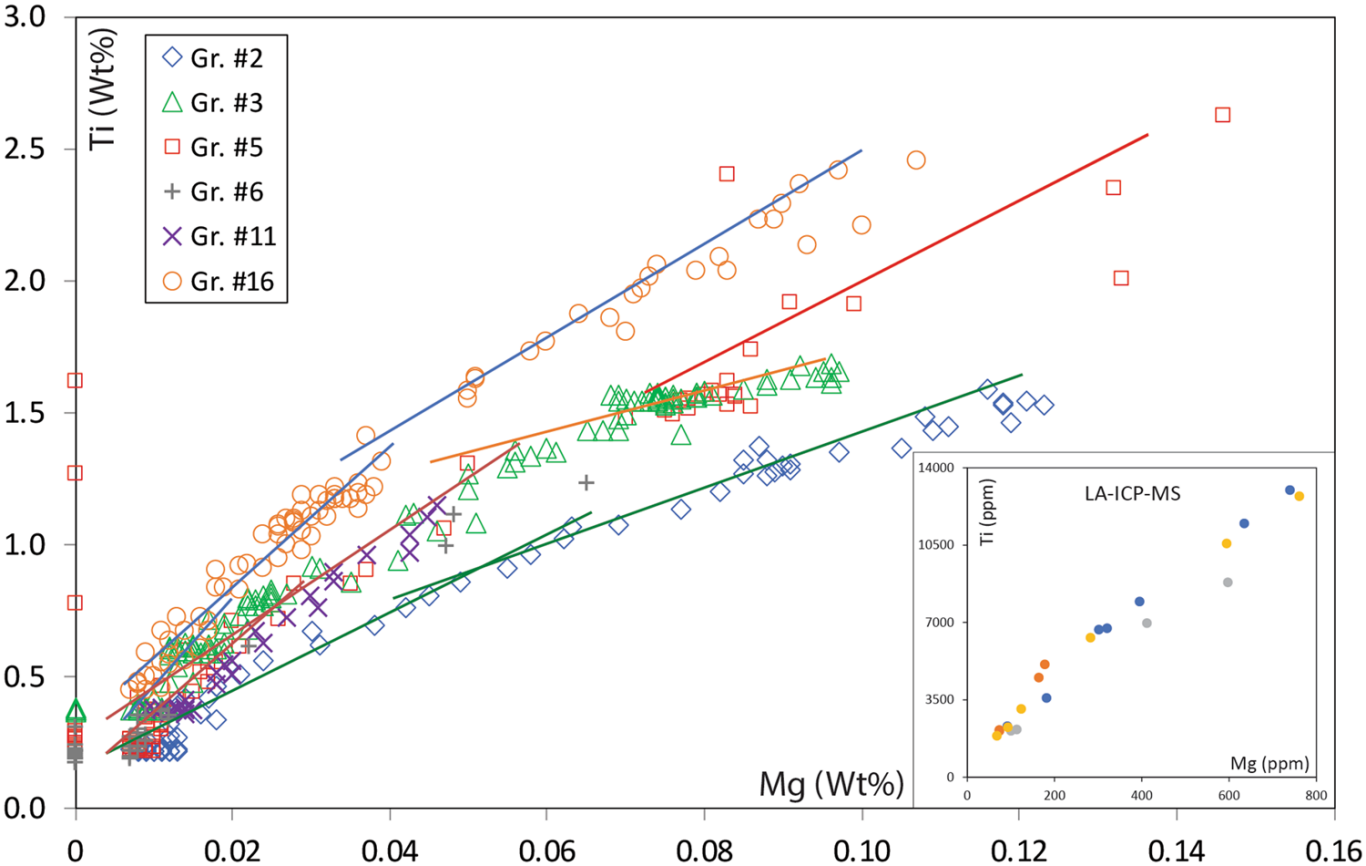
Plot of Ti vs. Mg (wt%, EMP data) in different grains of Carmel Sapphire; inset shows LAM-ICPMS data at lower concentrations.

Contents of Mg, the only other significant trace element, range up to 0.13 wt% (*ca* 0.08 at.%). Ti and Mg contents are closely correlated (Fig. 4), but the slope of the regression changes with Ti content. Within a single grain, Ti/Mg (atomic) lies at levels near 20 for lower Ti contents and decreases to values around 10 at higher Ti contents. LAM-ICPMS analyses (Table 1) show that the Ti-Mg correlation continues down to < 100 ppm Mg at 2000 ppm Ti. Positive correlations also are seen in plots of Sc and Zr vs. Ti; the plot of V vs. Ti shows a range of variants on this behaviour, from different grains (Fig. SD3). There is no correlation between the contents of Ti and Si.

**Table 1:** Analyses of glass in melt pockets, corundum, ruby and blue sapphire from Mt Carmel.

wt%	Glass^a^	ppm	Cor-05	Cor-04	Cor-02	Cor-06	Ruby	Sapphire	stdev
	n = 13		n = 4	n = 7	n = 4	n = 6	n = 16	n = 40	
	EMP		LA-ICPMS	LA-ICPMS	LA-ICPMS	LA-ICPMS	LA-ICPMS	LA-ICPMS	
SiO_2_	47.1	Mg	122	381	305	320	9.6	88	92
ZrO_2_	1.9	Si	1900	1770	1790	1670	1630	1480	131
ThO_2_	0.3	Sc	2.0	5.0	5.2	5.8	< 0.1	< 0.1	
TiO_2_	3.0	Ti	3460	7390	5010	6140	16	280	248
Al_2_O_3_	24.4	V	4.0	5.1	4.3	2.8	3.7	61	116
La_2_O_3_	0.4	Cr	0.1	3.3	3.8	0.7	10,700	200	517
Ce_2_O_3_	1.8	Mn	< 0.7	< 0.7	< 0.7	< 0.7	< 0.3	0.9	1.0
MgO	5.2	Fe	< 20	< 20	< 20	< 20	16	7270	2225
CaO	7.4	Co	< 0.1	< 0.1	< 0.1	< 0.1	0.02	0.1	0.1
Na_2_O	0.4	Cu	< 0.5	< 0.5	< 0.5	< 0.5	< 0.1	< 0.06	
K_2_O	1.7	Zn	< 0.7	0.30	< 0.7	0.4	1.6	1.0	1.3
SO_2_	1.6	Ga	< 0.2	< 0.2	< 0.2	< 0.2	35	155	66
Sum	95.3	Zr	4.4	14	14	13	0.2	0.5	1.8

^a^Mean of analyses of glass with no crystals other than tistarite.

### Numerical modelling of zoning

To understand how the unusual zoning patterns in the corundum are related to crystallization from the Al_2_O_3_-supersaturated melts, and the implications of the zoning for the growth environment, we have carried out numerical modelling of the zoning.

**Figure 5:**
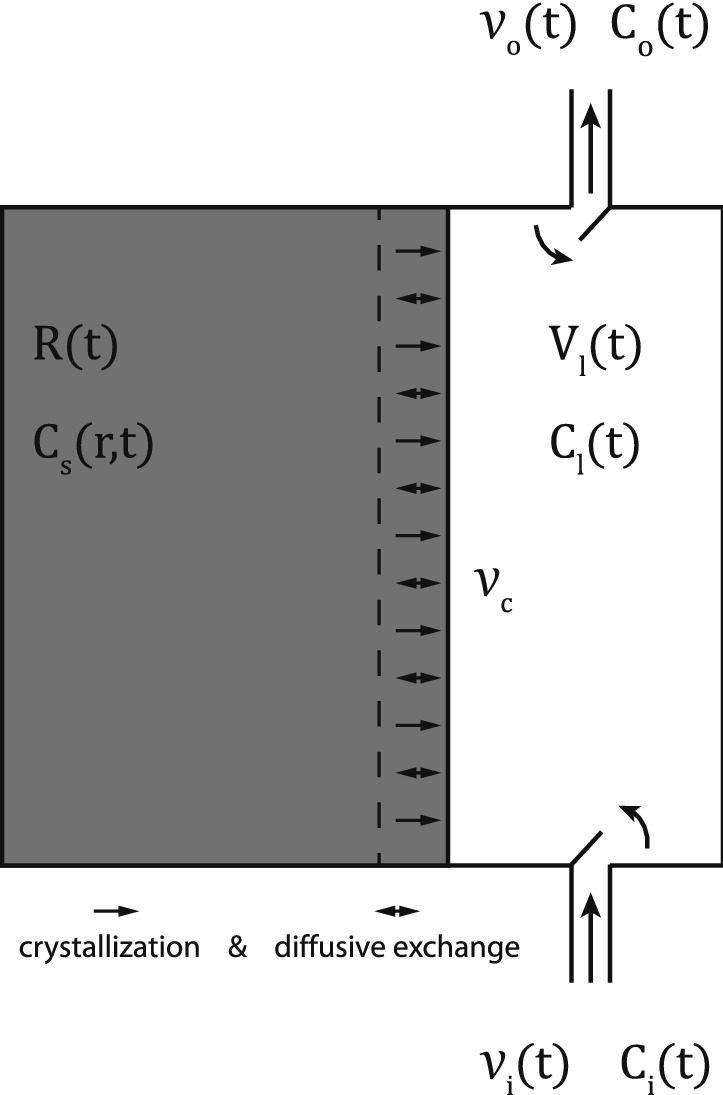
Schematic illustration of the model. Chemical component exchange between solid and melt occurs as a consequence of two processes: crystallization of the melt on the rim of the solid grain and because of diffusive exchange. The model considers both an inflow and outflow of melt in and out of the system, which may vary as the system evolves. The volume (per unit of surface) of corundum and melt are controlled by the crystallization (${v}_{c}$), inflow (${v}_{i}$) and outflow (${v}_{o}$) rates. Further details are given in the Appendix.

In our numerical simulations we consider a crystallization scenario involving two distinct Ti reservoirs: a corundum phase undergoing one-dimensional crystal growth, and a percolating melt (Fig. 5). This is modelled assuming that (a) the grain boundary is locally in equilibrium with the surrounding melt (i.e. the Ti content in the fluid and solid rim are linked by the partition coefficient); (b) the concentration of Ti at any point in the interior of the corundum can be modified only by chemical diffusion; (c) the melt phase is compositionally homogeneous; and (d) the volume of the melt reservoir changes with time as a result of variations in melt inflow (${v}_{i}$), melt outflow (${v}_{o}$), and crystallization (${v}_{c}$) (Fig. 5). These numerical simulations assume a constant crystallization rate of ${\alpha }_{c}={10}^{-7}$ cm/s, consistent with the rapid annealing of skeletal crystals observed in experiments^30^. Both inflow and outflow melt rates are considered to be proportional to the crystallization rate (i.e. ${v}_{o}={{\alpha }_{o}v}_{c}$ and ${v}_{i}={{\alpha }_{i}v}_{c}$, where ${\alpha }_{o}$ and ${\alpha }_{i}$ are two different proportionality constants). Diffusion coefficients are modelled according to the Arrhenius relation in^31^, with a fixed temperature of 1300 °C. The complete set of governing equations is given in the Appendix.

**Figure 6:**
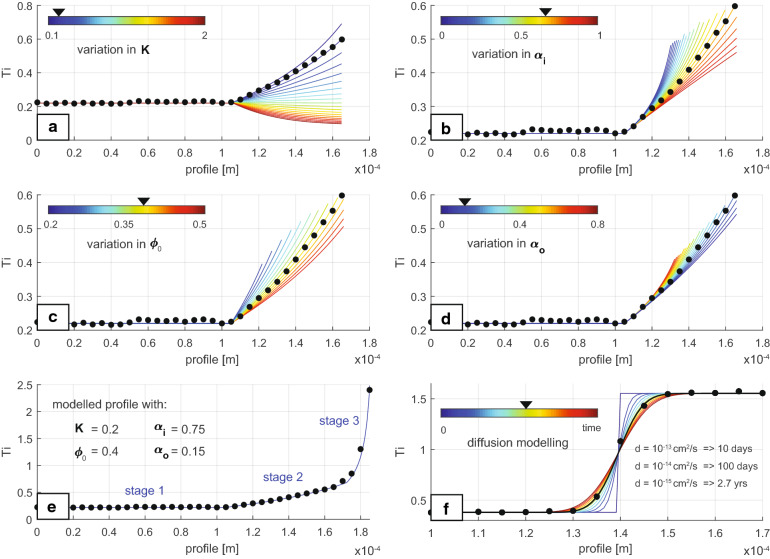
Results of modelling. (**a**–**d**) Show modelled corundum profiles during stage 2 for a range of partition coefficients ($K$), inflow rates (${v}_{i}$), initial melt content (${\phi }_{0}={V}_{l}(0)/(R(0)+{V}_{l}(0))$), and outflow rates (${v}_{o}$), respectively. Unless stated otherwise, all the models are run using reference values, $K=0.2; {\alpha }_{i}=0.75; {\phi }_{0}=0.4; {\alpha }_{o}=0.15$. The crystallization rate has been set to ${v}_{c}={10}^{-7}$ cm/s, following^30^, which implies that the crystallization takes ~ 1 day to complete. (**e**) Comparison between the modelled profile using the reference values and the 5-3 corundum traverse. (**f**) Diffusion modelling of a sharp concentration gradient using diffusion coefficients, $d={10}^{-13};{10}^{-14};{10}^{-15}$ cm^2^/s according to^31^. The times representing the best fit for each diffusion coefficient are shown in (**f**), and correspond to the highlighted green line. Other time values for each diffusion coefficient can be inferred relative to these highlighted values.

Under these assumptions, our numerical models show how the three different patterns observed in the Ti content within the corundum could be related to three different stages of a single crystallization process. Figure 6 compares results from our numerical model with corundum traverse 5-3 (Fig. 3).

Each of these three stages is characterized by a different set of ${\alpha }_{o}$ and ${\alpha }_{i}$ values. In the first stage, the observed nearly constant concentration of Ti within the corundum requires melts with constant Ti content to freely migrate through well- interconnected melt pathways (i.e. high values of melt inflow and outflow rates). In the second stage, the linear rise in Ti contents requires the free percolation of melts to be impeded (low rates of melt inflow and outflow). Figure 6a–d shows the effects on the corundum profiles of changing the main modelling parameters. Under specific conditions of initial melt abundance (~ 40% of melt), partition coefficient (K ~ 0.2), and melt inflow and outflow rates (${\alpha }_{o}=0.15$ and ${\alpha }_{i}=0.75$), our model is able to reproduce the observed nearly-linear rise of Ti in the corundum toward the melt pocket (Fig. 6e). As the crystals grow, melt pathways become narrower, eventually resulting in the formation of closed melt pockets (i.e. no percolation/extraction of melts from the system, ${\alpha }_{o}={\alpha }_{i}=0$). In this third stage, the content of incompatible elements in the melt rises exponentially (Fig. 6e). The fact that the transitions between these three stages are preserved within single corundum crystals indicates that diffusion processes are negligible compared with crystallization rates. This is true even when considering the effect of varying temperature in the diffusion model (note the jump of two to three orders of magnitude in diffusivities in Fig. 4 in^31^ or when using four to five orders of magnitude faster diffusivities from^32,33^).

Diffusion modelling of a sharp concentration gradient using a wide range of diffusivities (Fig. 6f) suggests that the whole crystallization process recorded in the corundum crystals occurred within days or a few years. Likewise, the crystal growth rate used in our crystallization experiments results in timescales of days for complete crystallization. Therefore, both diffusion and crystallization numerical experiments align with the observed timescales for annealing of skeletal crystals in experiments^30^.

## Discussion

Typical corundum megacrysts in alkali basalts contain < 0.1 wt% Ti^29^, and this raises the question of why Ti clearly substitutes readily into the Carmel Sapphire. The obvious answer would be that Ti is in the trivalent state, as is implied by the appearance of Ti_2_O_3_ and SiC in the melt pockets. The Ti^3+^ ion is larger than Ti^4+^ (ionic radius 0.67 vs. 0.605 Å in octahedral coordination^34^); both are considerably larger than Al^3+^ (0.53 Å), but the Al site in corundum is relatively spacious (<Al-O>  = 1.912 Å^35–37^), and substitution of Ti^3+^ does not require a charge-balance substitution.

The modelling provides insights into the processes that have led to such high levels of Ti^3+^ in the Mt Carmel corundum. The growth of aggregates of hopper/skeletal crystals is usually associated with nucleation on a substrate^38,39^. The rapid growth of skeletal or hopper crystals in a static situation would be expected to deplete the surrounding melt in compatible elements (in this case Al), while producing a zone enriched in incompatible elements. This would produce a zoning toward crystal rims enriched in Ti. The flat profiles of Ti vs distance in the early stages of corundum growth therefore suggest, on the contrary, that the melt was moving through the system rapidly enough to provide a continuous supply of “new” melt without significant enrichment in Ti, and still supersaturated in Al_2_O_3_; i.e. this was an open system. The most probable scenario may involve crystallization beginning on the walls of melt/fluid conduits, and that the system could remain open as long as the branching crystalline aggregate had a relatively large ratio of connected, permeable cavities to solid corundum (ca 40:60; Fig. 6c).

The modelling indicates that the first inflection point in the plots of Ti profiles (Fig. 3) represents a point in the growth of a given grain when fluid flow began to be restricted by the intersection of corundum crystals, and the more slowly-moving melts began to concentrate Ti more rapidly as Al_2_O_3_ was extracted. This inflection point also corresponds to a change from skeletal crystallization to a smoother zoning, suggesting a decrease in the corundum growth rate.

The second inflection point in some traverses, where the Ti-*vs*-distance profile changes from linear to exponential, would reflect the isolation of individual melt pockets from the external melt flow. This point corresponds to inflections in the Ti/Mg correlation profiles (Fig. 4). The level of Ti at the inflection points around different melt pockets may reflect differences in the composition of the melt at the time of closure of each pocket (Fig. 6b,d) or slightly different ratios of cavities to solid corundum (Fig. 6c). The drop in the Ti/Mg ratios at this point probably reflects the crystallization of tistarite (Ti_2_O_3_) from the trapped melts, while phenocrysts of Mg-bearing phases are rare. It is also possible that the distribution coefficient D_Ti_^cor/melt^ was changing with melt composition, but this is not required by the modelling.

The continued extraction of corundum from the trapped melt would lead to rapid concentration of incompatible elements (Ti, Zr, REE) in the residual melt, and in the coexisting corundum, and ultimately to the crystallization of a range of unusual minerals enriched in these elements^17,18^. The contents of incompatible elements such as Ti, Zr and Ce in the glasses increase sharply with decreasing Al_2_O_3_ (Fig. SD-4). This is generally consistent with a progressive reduction in melt volume, concentrating incompatible elements due to the fractional crystallization (growth) of corundum. The degree to which individual elements increase during this last stage depends strongly on the crystallisation of phases such as carmeltazite (ZrAl_2_Ti_4_O_11_); the concentration factors for elements such as Rb, Sr and HREE are relatively low (5–10) while concentrations of Th increase by a factor of > 60 during this third stage of crystallisation.

**Figure 7:**
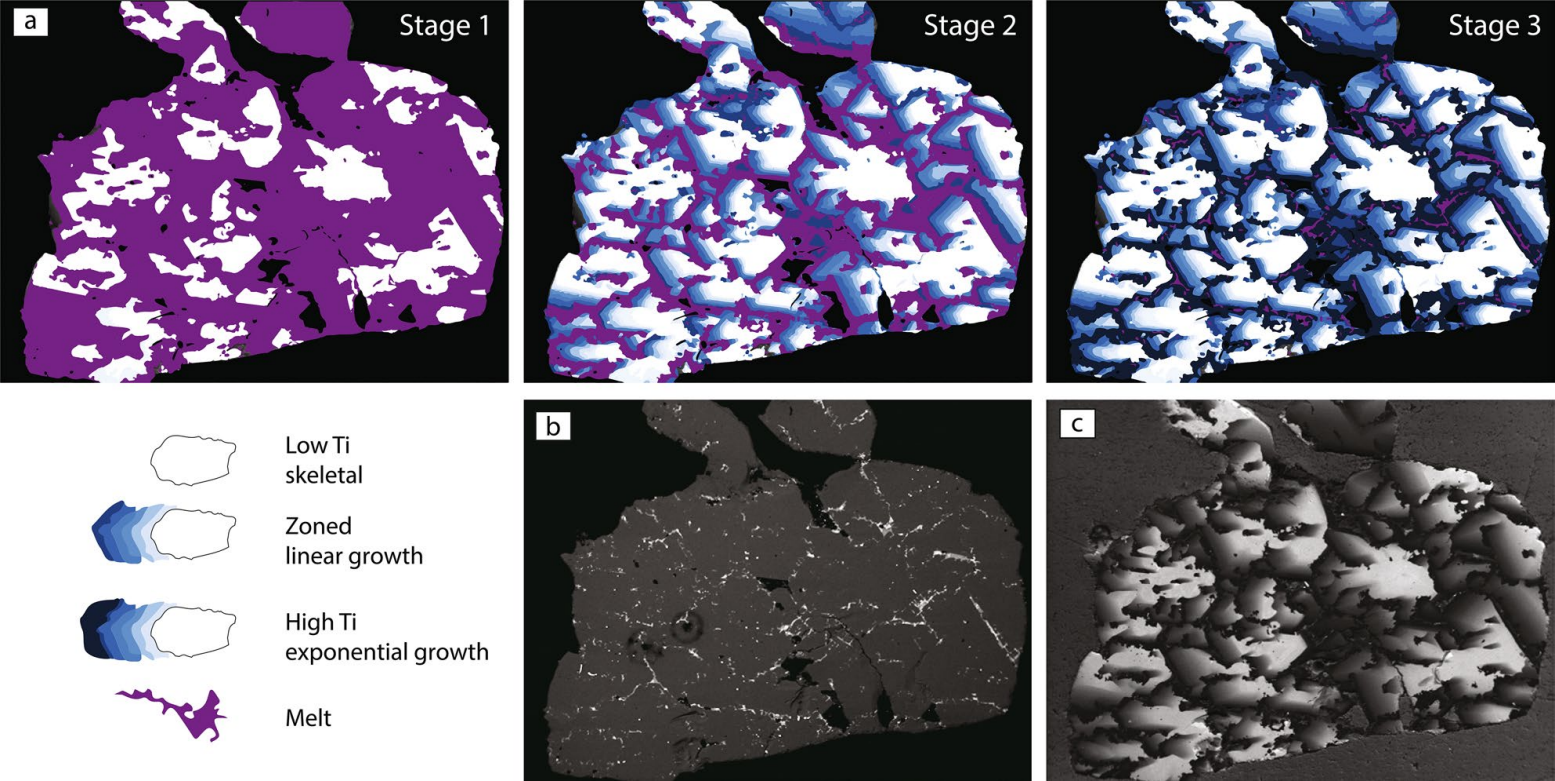
(**a**) Cartoon showing the distribution of corundum crystallized during the different stages of growth, in a crystal aggregate cut roughly subparallel to the C axes of the corundum. Aggregate is 2 mm long. (**b**) BSE image in which corundum is dark grey, void spaces are black, and melt pockets are highlighted by white crystals in grey glass. (**c**) Cathodoluminescence image (real colour of light areas is bright pink). In some white areas it is possible to see very skeletal structures, some surrounded by hopper walls with oscillatory zoning, perhaps suggesting relatively rapid crystallisation.

Figure 7 maps the distribution of corundum crystallized during the different stages of growth, in a crystal aggregate cut approximately subparallel to the [0001] axes of most crystals. It shows the very open structure produced during open-system crystallisation, the progressive closure of the system produced by rapid growth, and the small volume of material crystallized in the closed system around each trapped melt pocket. The strong sense of directional growth in the CL zoning suggests growth from a substrate, either a “floor” or a “wall”, toward the moving melt/fluid.

The implied rapid growth of the corundum crystals, and the short-range diffusion profiles at sharp contacts between high- and low-Ti volumes (Fig. 3) suggest that the melt system crystallising the Carmel Sapphire was very short-lived, especially on a geological time-scale (Fig. 6f). It was terminated by explosive eruption, producing large-scale brecciation; shattered aggregates of corundum crystals are cut by numerous veins of amorphous carbon, providing further evidence of the vital role of reduced COH fluids in this system.

These conclusions are robust despite the uncertainties in the diffusion coefficients, which may vary 3–4 orders of magnitude between studies^31^. Slow diffusivities of the order of ${10}^{-15}$ cm^2^/s result in a time scale of few years, whereas a timescale of days is obtained when using faster diffusivities (e.g. ${10}^{-12}$ cm^2^/s). However, when assuming an average corundum growth rate of ${10}^{-7}$ cm/s^30^, our traverse modelling estimates that complete crystallization is achieved in less than an hour. Therefore, if both processes (i.e. corundum crystal growth and diffusion of sharp concentration gradient) are to record the same timescale, these modelling results favour the use of faster diffusivities (of the order of ${10}^{-11}$ cm^2^/s^32,33^ or slightly slower crystal growth rates than the ones estimated in^31^ (on the order of ${10}^{-9}$ cm/s).

It might be argued that reducing fluids cannot exist in the lithospheric mantle because they would be oxidized by contact with normal (FeO-bearing) mantle rocks. Our modelling indicates short time-scales and the potential coating of the walls of fluid conduits by early-crystallized phases (in this case corundum); both factors offer possible solutions to the problem of generating and preserving extreme local *f*O_2_ heterogeneity in the mantle.

### Broader significance

The discovery of tistarite (Ti_2_O_3_) in the Mt Carmel material was the first report of a terrestrial Ti^3+^ mineral, and the Mt Carmel examples represent, to our knowledge, the first recognition of Ti^3+^ substitution in natural corundum^17^. These are not simply interesting mineralogical curiosities; their terrestrial occurrence has a wider significance. As noted above, the *f*O_2_-T conditions required to produce SiC and Ti^3+^-bearing phases would not be expected to occur in Earth’s upper mantle. Their presence therefore suggests that mantle-derived melts may be accompanied by deep-seated CH_4_–H_2_ fluids; when concentrated in specific environments at shallower depths such fluids clearly can produce at least transient volumes of highly-reduced rocks. An important question is: how widespread are these processes, and what is their role in the global carbon cycle?

Xiong et al.^18^ have reviewed a number of examples of similar highly-reduced assemblages, briefly summarized here. In chromitites in ophiolites from Tibetan and Polar Urals, Ti-corundum occurs together with SiC, diamond, alloys and other phases indicative of low *f*O_2_, many included in the corundum^11^. Explosive volcanic eruptions on the Kamchatka Peninsula carry a xenocryst assemblage that includes native Fe and Al, SiC and Ti-corundum^40,41^. A corundum inclusion with 1.8 wt% Ti was found in a diamond from the Monastery Mine kimberlite (South Africa)^42^; other diamonds from this mine contain inclusions of SiC. Similarly, Otter and Gurney^43^ reported two corundum inclusions, with 0.7 and 1.2 wt% Ti, in diamonds from the Sloan kimberlite in Colorado, USA.

It therefore seems that localized volumes of highly-reduced mineral assemblages may commonly develop in the lithospheric mantle, through the introduction of mantle-derived, magma-transported CH_4_–H_2_ fluids. The Mt Carmel example implies that these localized reduced volumes can be protected from oxidation by the surrounding mantle by crystallization of phases like corundum. The sampled Mt Carmel systems were transient, and destroyed by eruption of the host magma; others might survive where eruption did not occur. The interaction of such reduced fluids with the lithospheric mantle may also be the ultimate source of more oxidized metasomatic fluids dominated by CO_2_ + H_2_O.

## Conclusions

Numerical modelling of Ti^3+^ zoning patterns traces three stages of magmatic crystal growth in corundum aggregates (“Carmel Sapphire”) found as xenoliths in Cretaceous pyroclastic rocks on Mt Carmel, N. Israel. (1) Crystallisation of skeletal/hopper crystals began on a substrate and continued in an open system (flowing melt) at *f*O_2_ = ca ΔIW-6. (2) As further crystal growth reduced porosity and impeded fluid flow, Ti^3+^ concentrations in the corundum rose linearly. (3) Crystal growth finally isolated individual pockets of the melt in a 3D network, and Ti^3+^ concentrations in both melt and corundum rose exponentially as fractional crystallisation of corundum reduced melt volumes. Ti remained an incompatible element during crystallisation even when concentrations in corundum exceeded 2 wt%, as did other elements such as Mg, Zr and REE. Modelling of diffusion profiles at sharp contacts near melt pockets indicate that the entire crystallization process took place on timescales of days to years. The presence of glass in the melt pockets indicates that crystallization was ongoing immediately before the explosive eruption of the host magma; this may suggest a genetic connection between that magma and the CH_4_-H_2_ dominated fluids responsible for the highly reduced conditions. Ti-bearing corundum may be used as an indicator of these conditions in the lithospheric mantle.

## Methods

### Sampling

The sampling methods used in the field and laboratory, designed to avoid external contamination, have been described in detail elsewhere^22^.

### Analytical techniques

#### SEM, EMP

Samples were mounted in epoxy blocks, polished and coated with carbon. A Zeiss EVO MA15 scanning electron microscope (SEM) at the Geochemical Analysis Unit (GAU), Macquarie University, Sydney, Australia^27,28^ was used to capture Backscattered Electron (BSE) images. Energy Dispersive X-ray spectrometry (EDS) was used to map the elemental composition of the samples, and to analyze phases of interest. An accelerating voltage of 15 keV and a beam current of 1 nA was used.

Major and minor elements were determined by electron microprobe (EMP) using a CAMECA SX100 equipped with five wavelength-dispersive spectrometers at the Macquarie University GeoAnalytical (MQGA; formerly GAU), Macquarie University, Sydney, Australia^27^. Analyses were performed using a focused beam (1–2 µm) with an accelerating voltage of 15 keV and a beam current of 20 nA. Standards were a suite of natural and synthetic minerals; specifically Al_2_O_3_ for Al, TiO_2_ for Ti and MgO for Mg. Peak counting varied between 10 and 20 s, and background was counted for 5–10 s on either side of the peak. Oxygen and carbon were measured directly; matrix corrections were carried out by the ZAF software.

Major and minor elements also were determined by electron microprobe (EMP) using a JEOL 8530F electron microprobe equipped with five wavelength dispersive spectrometers at the Centre for Microscopy Characterisation and Analysis (CMCA), The University of Western Australia, Perth, Australia^27^. Analyses were preformed using a fully focussed beam with an accelerating voltage of 15 keV, a beam current of 15 nA. Standards were a suite of natural and synthetic minerals. On-peak counting times were 30 s for all elements. X-ray intensity data was corrected for Time Dependent Intensity (TDI) loss (or gain) using a self-calibrated correction for Si kα, Ti kα, Mn kα, Na kα, K kα and mean atomic number (MAN) background corrections were used throughout. Unknown and standard intensities were corrected for deadtime. Oxygen was measured, matrix corrections and ZAF correction were applied throughout.

#### Trace element analysis

Trace elements were obtained using a Photon Machines Excite 193 nm ArF EXCIMER laser system coupled to an Agilent 7700 × ICP-MS at the GAU, Macquarie University, Sydney, Australia^27^. Detailed method descriptions have been given by^44^. The ablation conditions included beam size (50 μm), pulse rate (5 Hz) and energy density (7.59 J/cm^2^). Ablation was performed in an HelEx II cell and helium was used as the carrier gas at a total flow rate of 0.825 L/min. Average analytical runs comprised of 20 analyses, 2 analyses of reference materials and 10 analyses of unknowns, bracketed by two analyses of NIST610 standard glass at the beginning and end of each run. Analyses consisted of 60 s of background and 120 s of ablation. Trace element concentrations were calculated from the raw signal data using the on-line software package GLITTER (http://www.mq.edu.au/GEMOC). GLITTER calculates the element concentrations and relevant isotopic ratios for each mass sweep and displays them as time-resolved data. This allows homogeneous segments of the signal to be selected for integration. GLITTER then corrects the integrated ratios for ablation-related fractionation and instrumental mass bias by calibration of each selected time segment against the identical time-segments of the standard glass (NIST610 using the GeoReM values). An appropriate major element was used as an internal standard to quantify trace element concentrations and the synthetic BCR2 and NIST612 glasses were used as reference materials (GeoReM).

#### Transmission electron microscopy

FIB foils from the region of interest were prepared for TEM using a dual‐beam FIB system (FEI Helios G3CX). High Angle Annular Dark Field Scanning Transmission Electron Microscopy (HAADF‐STEM) imaging and element mapping were carried out using an FEI Titan G2 80‐200 TEM/STEM with ChemiSTEM Technology operating at 200 kV at the Centre for Microscopy Characterisation and Analysis (CMCA), The University of Western Australia, Perth, Australia^27,28^. The element maps were obtained by energy dispersive X‐ray spectroscopy using the Super‐X detector on the Titan with a probe size ~ 1 nm and a probe current of ~ 0.25 nA. Total acquisition times of 20–30 min were used to obtain good signal‐to‐noise ratios. Electron diffraction was carried out using a field limiting aperture that selected an area approximately 400 nm in diameter.

## Supplementary Information

Supplementary Figures

Supplementary Table S1.

Supplementary Appendix S1.

## Data Availability

All data used in this paper are contained in the Supplementary Information.
