# Supplementary material for: Ti3+ in corundum traces crystal growth in a highly reduced magma
Source: Sci Rep. 2021 Jan 28;11:2439. doi: 10.1038/s41598-020-79739-4 (PMC7844248; doi:10.1038/s41598-020-79739-4)
Supplement: Supplementary file 1 — Supplementary Figures [file 41598_2020_79739_MOESM1_ESM.docx]

Supplementary Figures


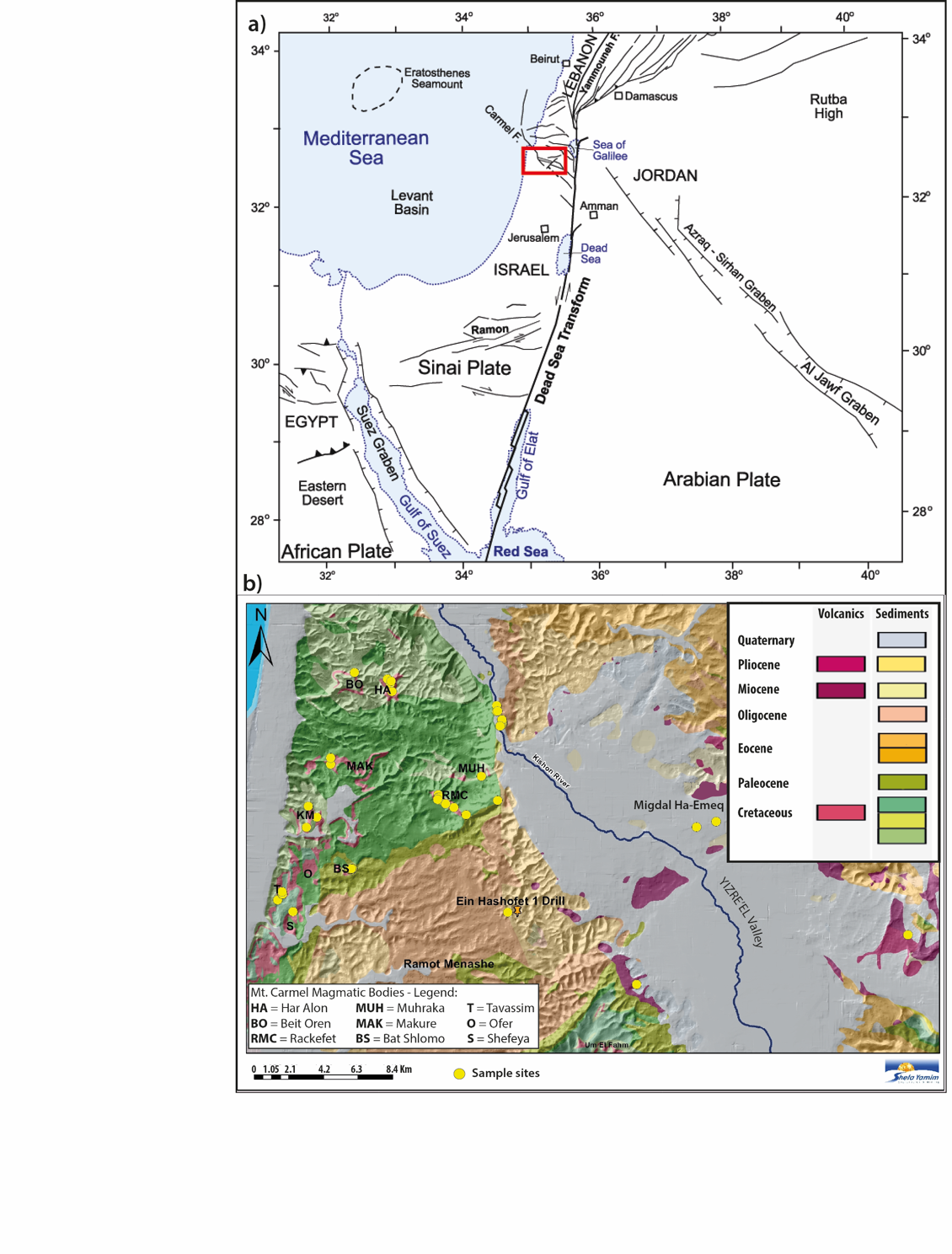


Figure SD-1. Location map of study area, with simplified geological map and locations of Cretaceous volcanic centers. (a) modified from [20]; (b) modified from [47].


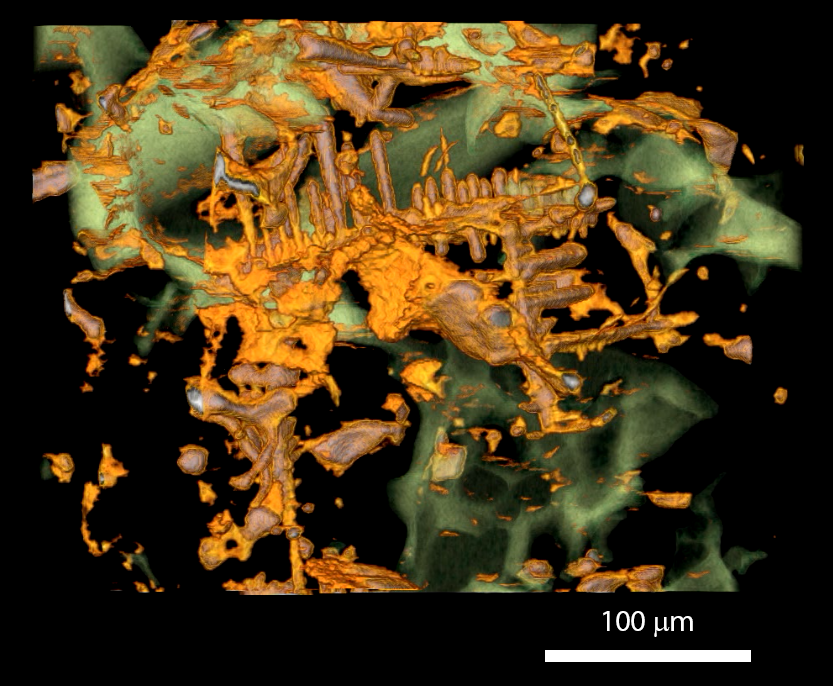


Fig. SD2. 3D-µCT image of a section of a Carmel Sapphire aggregate. Corundum is shown in black; green volumes are voids, presumably fluid-filled; orange to white colours show increasing density, with highest densities representing grains of Fe-Ti-Si alloys and skeletal cavities usually filled with Ti(N,O).


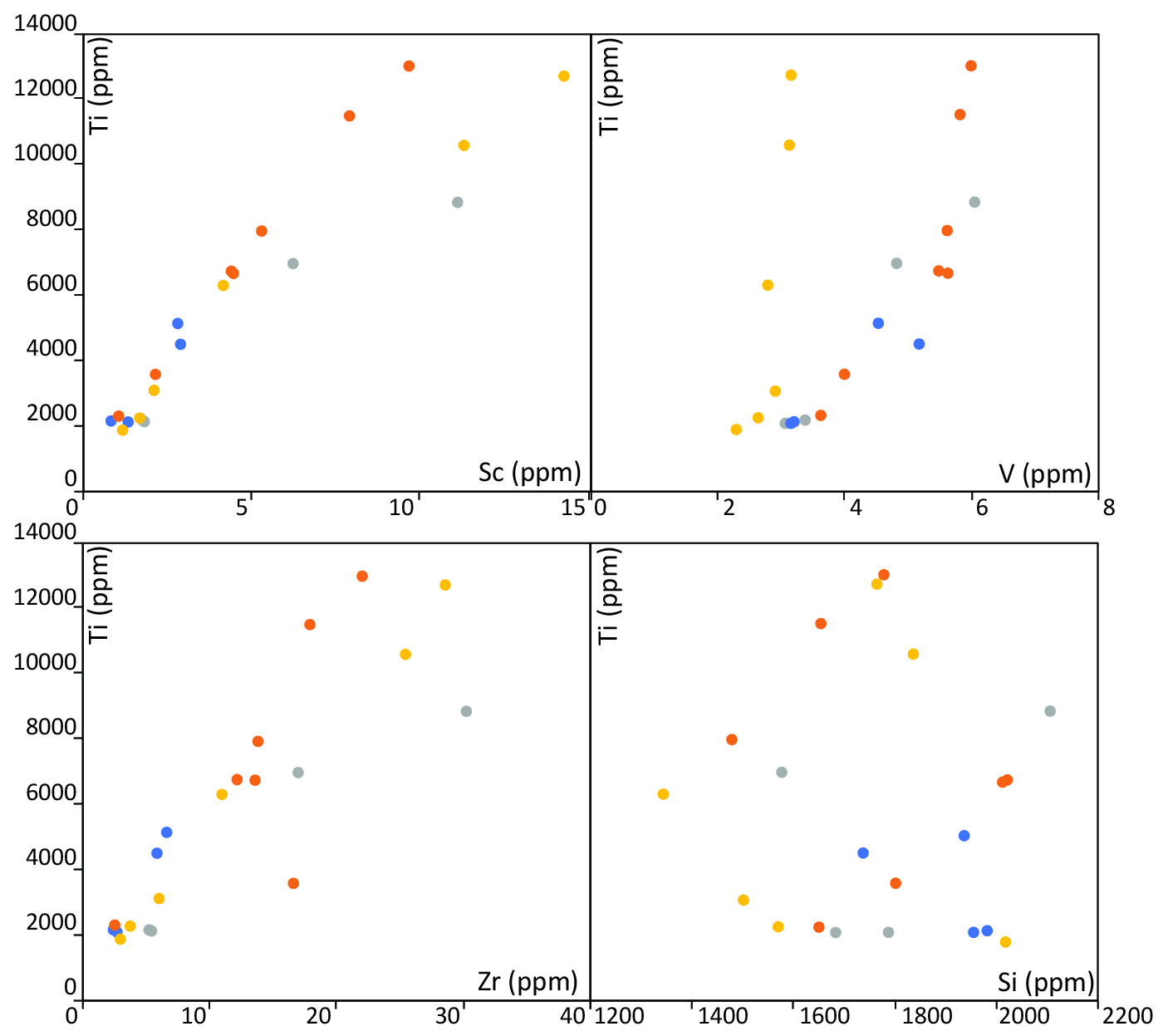


Fig. SD-3. LAM-ICPMS analyses of corundum grains with different Ti levels. Coloured spots denote analyses within individual grains.


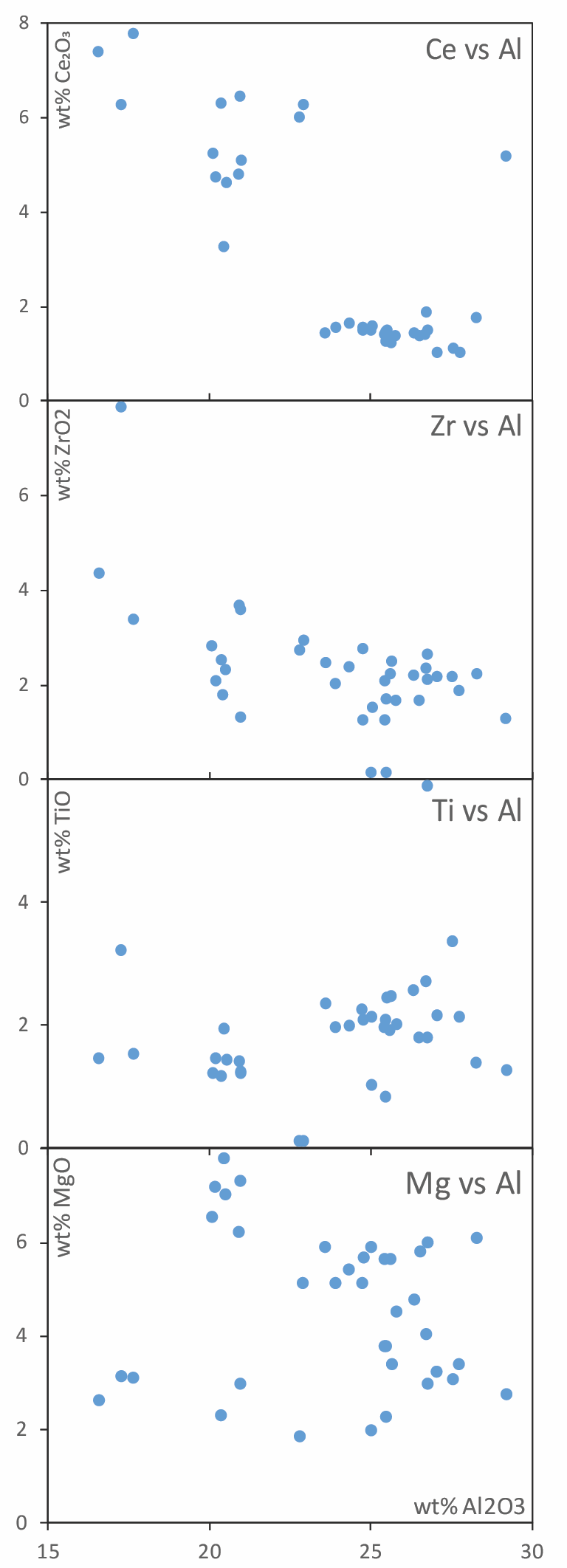


Fig. SD-4. Ti, Mg, Zr, Ce *vs* Al_2_O_3_ in residual glasses of melt pockets
