# Supplementary material for: Ti3+ in corundum traces crystal growth in a highly reduced magma
Source: Sci Rep. 2021 Jan 28;11:2439. doi: 10.1038/s41598-020-79739-4 (PMC7844248; doi:10.1038/s41598-020-79739-4)
Supplement: Supplementary file 3 — Supplementary Appendix S1. [file 41598_2020_79739_MOESM3_ESM.docx]

**Appendix. Description of the model**

We model one-dimensional mineral growth of a corundum crystal, where chemical exchange of Ti is allowed between the crystallizing corundum and the percolating melt. A schematic illustration of the model is shown in Figure 5. Both the length of the corundum crystal $R\left( t \right)$ and melt volume $V_{l}\left( t \right)$ evolve with time as crystallization proceeds. For this model the following is assumed:

(a) the grain-boundary is locally in equilibrium with the surrounding melt (i.e. Ti content in the fluid and solid rim are linked by the partition coefficient).

(b) the concentration of a Ti at any point within the corundum can be modified only by chemical diffusion.

(c) that the melt phase is compositionally homogeneous.

(d) the volume of the melt reservoir changes with time as a result of melt inflow ($v_{i}$), melt outflow ($v_{o}$), and crystallization ($v_{c}$)

Under these assumptions, the concentration of Ti in the corundum can be described by the following diffusion equation along the growing axis ($r$):

$\frac{dC_{s}(r,t)}{dt}=D\frac{d^{2}C_{s}(r,t)}{dr^{2}}, 0\leq r\leq R(t)$ (1)

with initial condition

$C_{s}\left( r,0 \right)=C_{s}^{0}$ (2)

and boundary conditions

$C_{s}\left( R,t \right)=KC_{l}(t)$ (3)

$C_{s}\left( 0,t \right)=C_{s}^{0}$ (4)

In these equations, $C_{s}(r,t)$ corresponds to the concentration of Ti along the $r$-direction of the crystal growth; $R(t)$ is the representative size of the corundum in $\left[ m \right]$ in the $r$-direction; $K$ refers to the partition coefficient; and $D$ is the diffusion coefficient in $\left[ m^{2}/s \right]$.

The Ti concentration in the melt is described by,

$\frac{d}{dt}({V_{l}\left( t \right)C}_{l}(t))=-\frac{d}{dt}\left\{ \int_{o}^{r} C_{s}\left( r,t \right)dr \right\}-v_{o}C_{o}+v_{i}C_{i}$ (5)

where $V_{l}$ refers to the melt volume in $\left[ m^{3}/m^{2} \right]$, $v_{i}$ and $v_{o}$ refer to the melt inflow and outflow rate in $\left[ m/s \right]$, and $C_{l}$ and $C_{o}$ their respective compositions. Equation (5) is a mass conservation equation for Ti content in the melt, which states that the content of the residual melt is determined by the total Ti content gained by the corundum due to both diffusion and crystallization (first term in the right-hand-side, (RHS)), the extraction of melt out of the system (second term in the RHS), and the melt input into the reservoir (last term in the RHS). In this work we assume that the extracted melt is compositionally equal to the melt within the system, and thus ${C_{o}=C}_{l}$. Also, we consider an inflow of melt entering the system with a constant composition, i.e. ${C_{i}=C}_{l}(0)$.

We model the evolution of both corundum grain size, $R\left( t \right),$ and melt volume $V_{l}\left( t \right)$ as a linear function of crystal growth rate $v_{c}$, and melt inflow and outflow rates $v_{i}$ and $v_{o}$,

$R\left( t \right)=R\left( 0 \right)+v_{c}t$ (6)

$V_{l}\left( t \right)=V_{l}\left( 0 \right)+\left( v_{i}-v_{o}-v_{c} \right)t$ (7)

The system of Eqs. 1-7 is solvable provided that crystal growth rate, $v_{c}$, melt outflow rate, $v_{o}$, and melt inflow rate, $v_{i}$, are known. Alternatively, based on [45] one can define melt outflow and inflow rates as $v_{o}={\alpha_{o}v}_{c}$, and $v_{i}={\alpha_{i}v}_{c}$, where $\alpha_{o}$ and $\alpha_{i}$are the ratios ratio of outflow and inflow rates over growth rate, respectively. In the case of a closed system, or a very inefficient melt transport mechanism (i.e. $\alpha_{o}= \alpha_{i}=0$), our model describes equilibrium crystallization, whereas fractional crystallization is recovered when the melt is only allowed to be extracted (i.e. $\alpha_{0}=1$ and $\alpha_{i}=0$). Equation 7 implies that in order to obtain a decrease in the melt volume of the system the second term in the right-hand-side has to be negative. Or in simple terms, that the melt inflow has to be smaller than the sum of crystallizing and melt outflow rates.

In this work we explore the effects and implications of choosing different values of $\alpha_{o}$ and $\alpha_{i}$ in an attempt to infer the mechanical and chemical conditions that lead to the corundum formation with time.

References

[46] Qin, Z. Disequilibrium partial melting model and its implications for trace element fractionations during mantle melting. *Earth Planetary Science Letters* **112**, 75-90 (1992).

[47] Hattingh, J. Updated Competent Person’s Report on the Shefa Gems Ltd Gemstone Assets. https://[www.shefagems.com/cpr](http://www.shefagems.com/cpr). 114 pp. (2019).
